# 肿瘤微环境中脂肪细胞对肺腺癌A549细胞生物学行为影响的初步研究

**DOI:** 10.3779/j.issn.1009-3419.2018.05.01

**Published:** 2018-05-20

**Authors:** 航 张, 晶晶 李, 亚男 曹, 翔 董, 聪 高, 烦繁 李

**Affiliations:** 230601 合肥，安徽医科大学第二附属医院肿瘤科 Department of Oncology, the Second Affiliated Hospital of Anhui Medical University, Hefei 230601, China

**Keywords:** 肺肿瘤, 脂肪细胞, 细胞增殖, 迁移, 侵袭, Lung neoplasms, Adipocyte, Cell proliferation, Migration, Invasion

## Abstract

**背景与目的:**

脂肪细胞在肿瘤微环境中可通过提供代谢燃料或信号传导媒介等方式以促进多种恶性肿瘤细胞的增殖与侵袭，但其在肺癌进展过程中的作用尚不清楚。本研究旨在探讨脂肪细胞对肺癌细胞生物学行为的影响。

**方法:**

将3T3-L1前脂肪细胞诱导为成熟脂肪细胞；采用显微镜成像、油红O染色试验观察细胞形态；应用MTT、平板克隆形成实验、划痕实验以及Transwell检测肺癌细胞增殖、迁移及侵袭能力的变化；通过比色分析法测定细胞内甘油三酯的含量。

**结果:**

与成熟脂肪细胞共培养后，肺腺癌A549细胞的形态变得更加纤长，增殖与克隆形成能力明显增强（*P* < 0.05）；此外，成熟脂肪细胞亦可促进A549细胞的迁移（*P* < 0.05）、侵袭（*P* < 0.01）以及胞内脂质的累积（*P* < 0.05）。

**结论:**

肿瘤微环境中的脂肪细胞可促进肺腺癌A549细胞的增殖、迁移与侵袭，且这一促进作用可能与脂质代谢相关。

## Abstract (en)

**Background and objective:**

Adipocytes in the tumor microenvironment may provide the metabolic fuel or signal transduction through media and other means to promote a variety of malignant proliferation and invasion, of tumor cells, but their role in lung cancer progression is still unclear. The purpose of this study was to investigate the effect of adipocytes on lung cancer cell biology.

**Methods:**

3T3-L1 pre-adipocytes were induced into mature adipocytes. The cell morphology was observed by microscopy and Oil Red O staining. MTT assay, colony formation assay, wound-healing and Transwell methods were used to detect lung cancer cell proliferation, migration and invasion ability. The content of triglyceride in cells was determined by colorimetry.

**Results:**

The morphology of lung adenocarcinoma A549 cells became more slender after co-culture with mature adipocytes, and the proliferation and cloning ability were significantly enhanced (*P* < 0.05). In addition, mature adipocytes can also promote the migration ability (*P* < 0.05), invasion ability (*P* < 0.01) and accumulation of intracellular lipid (*P* < 0.05) of A549 cells.

**Conclusion:**

These findings suggested that adipocytes in tumor microenvironment can promote the proliferation, migration and invasion of lung adenocarcinoma A549 cells, which may be related to lipid metabolism.

肺癌是世界范围内发病率与死亡率最高的恶性肿瘤^[1]^。由于没有特异性症状与体征，大部分肺癌患者在诊断阶段已发生远处转移，其诊断后5年生存率不到20%^[2]^。因此，对肺癌相关危险因素的研究以及对其发病机制的深入了解在肺癌的防治中有着重要意义。脂肪组织及其相关代谢异常性疾病，近年已在流行病学与基础性研究中被确定为某些恶性肿瘤的不良预后因素^[3, 4]^。作为脂肪组织的主要组成成分，脂肪细胞已被应用于多种癌症的研究之中，包括前列腺癌、胰腺癌、卵巢癌、乳腺癌和黑色素瘤等，其通过提供代谢燃料或信号传导媒介等方式以促进肿瘤细胞的增殖与侵袭^[5-9]^。然而，脂肪细胞在肺癌进展过程中的作用尚未被证实。虽然以往一般认为肺癌不是肥胖相关性癌症，但高脂饮食（HFD）所诱发的超重或肥胖症增加了小鼠肺癌形成率与癌症相关死亡率^[10, 11]^。此外，骨（骨髓脂肪细胞是骨髓微环境中数量最多的细胞类型；且随着年龄的增加，其在骨髓中的比例不断增高^[12]^）、脑（60%由脂质组成）以及肝脏（机体内主要的脂质代谢器官）是肺癌的主要转移部位，根据“种子与土壤”理论^[13]^，我们应考虑到脂肪细胞及其代谢产物在肺癌转移过程中的作用。本研究旨在探索脂肪细胞对肺腺癌A549细胞生物学行为的影响，并对其相关机制进行初步探讨。

## 1 材料与方法

### 1.1 细胞及试剂

A549人肺腺癌细胞株与3T3-L1前脂肪细胞株由上海复旦大学惠赠。DMEM高糖培养基购自美国HyClone公司；新生牛血清，胎牛血清购自杭州四季青生物工程材料有限公司；油红O染料购自生工生物工程（上海）股份有限公司（进口分装）；青霉素/链霉素购自美国Gibco公司；地塞米松与3-异丁基-1-甲基黄嘌呤（IBMX）购自美国Sigma公司；牛胰岛素购自上海索莱宝公司；结晶紫、溴化-3（4, 5-二甲基噻唑基-2）-2, 5-二苯基四唑（MTT）与牛血清白蛋白购自美国Amresco公司；甘油三酯（Triglyceride, TG）测定试剂盒（A110-1）购自南京建成生物工程研究所；Transwell（0.4 μm，8 μm孔径）小室购自美国Corning公司；Matrigel胶购自美国BD公司。

### 1.2 细胞培养及3T3-L1细胞的诱导分化

用含10%胎牛血清、1%青霉素/链霉素的DMEM完全培养基和含10%新生牛血清、1%青霉素/链霉素的DMEM完全培养基，在37 ℃、5%CO_2_的细胞培养箱中分别培养A549细胞与3T3-L1细胞。待3T3-L1细胞生长至接触抑制2天后开始诱导分化：细胞首先培养于诱导剂1（在含10%胎牛血清的DMEM中加入10 μg/mL胰岛素，1 μM/L地塞米松和0.5 mM/L IBMX）。两天后，培养液替换成诱导剂2（在含10%胎牛血清的DMEM中加入10 μg/mL胰岛素），并继续培养2天。随后每两天更换一次培养基。待培养至第8天时，大约80%的前脂肪细胞可分化为成熟脂肪细胞。

### 1.3 油红O染色

用PBS洗涤成熟脂肪细胞或A549细胞两次，4%多聚甲醛固定20 min，再用PBS清洗2遍后，每孔加入0.6 mL油红O染液染色20 min，弃去染液，用60%异丙醇洗涤至背景清晰后，在光学显微镜下观察细胞。

### 1.4 Transwell间接共培养及实验分组

采用Transwell共培养系统（0.4 μm孔径；Corning，USA），将前脂肪细胞和成熟脂肪细胞分别与A549细胞间接共培养：取对数生长期A549细胞铺于6孔板或24孔板中（即下室），前脂肪细胞或成熟脂肪细胞铺于Transwell小室中（即上室）。实验分组如下：Group 1（A549细胞单独培养，即空白对照组）；Group 2（A549细胞与3T3-L1前脂肪细胞共培养，即前脂肪细胞共培养组）；Group 3（A549细胞与成熟脂肪细胞共培养，即成熟脂肪细胞共培养组）。

### 1.5 MTT法

每组设5个平行孔。用无血清DMEM培养基将A549细胞密度调整为1.2×10^4^/孔，取600 μL细胞悬液置于24孔板中，培养12 h后待其贴壁（此时计为Day 0）。吸弃旧培养基，应用Transwell小室（0.4 μm）将3T3-L1前脂肪细胞、成熟脂肪细胞分别与A549细胞间接共培养1天-3天后，将小室取出，在铺有A549细胞的24孔板中每孔避光加入60 μL MTT（5 mg/mL），继续培养4 h后终止培养。弃去孔内旧培养液，每孔加入600 μL DMSO，摇床上振荡混匀10 min使甲瓒结晶充分溶解。分别吸取150 μL/孔溶解物至96孔板，用酶标仪检测波长490 nm处各孔的吸光度（OD）值，以OD值来表示细胞的增殖活力。

### 1.6 平板克隆形成实验

A549细胞以1, 000/孔接种于6孔板，待其贴壁后，将铺有前脂肪细胞或成熟脂肪细胞的小室悬挂于培养有A549细胞的6孔板上，上下室叠放开始间接共培养。2周后，移去小室，PBS清洗2次，4%多聚甲醛固定20 min，随后加入0.1%结晶紫染液染色15 min并置于显微镜下观察计数。

### 1.7 细胞划痕实验

接种A549细胞于6孔板中，待细胞融合度达80%以上时，用200 μL移液枪枪头沿直尺，并垂直于6孔板底面均匀划3道横线，PBS洗涤两次后，将铺有前脂肪细胞或成熟脂肪细胞的小室悬挂于培养有A549细胞的6孔板上，间接共培养于含1%牛血清白蛋白的DMEM中。分别于0 h、24 h在倒置光学显微镜下拍摄照片，并测量划痕区域面积的变化。

### 1.8 Transwell迁移与侵袭实验

与前脂肪细胞或成熟脂肪细胞共培养2天的A549细胞经胰酶消化及离心后，用无血清DMEM培养基重悬并调整细胞密度为1×10^5^/mL，将200 μL细胞悬液加入Transwell（8 μm）上室；下室加入600 μL含10%胎牛血清的DMEM培养液。继续培养24 h，取出Transwell小室，用4%多聚甲醛固定，0.1%结晶紫染色，镜下拍照并统计穿膜细胞数，以此来反映A549细胞迁移能力。侵袭实验除去向Transwell小室加入细胞悬液之前，在小室内面铺一层50 μg/mL Matrigel胶，其余实验步骤基本与Transwell迁移实验相同。

### 1.9 胞内甘油三酯检测

肺腺癌A549细胞以1×10^5^/mL的密度接种于6孔板中，待其贴壁后，将铺有前脂肪细胞或成熟脂肪细胞的小室悬挂于6孔板上。共培养48 h后，移去上室，消化下A549细胞并经超声破碎处理后，使用甘油三酯测定试剂盒检测细胞内甘油三酯的含量。

### 1.10 统计学处理

实验结果的统计分析均用SPSS 13.0和GraphPadPrism 7进行。所有实验均独立重复3次，数据以Mean±SD表示，多组间均数比较采用单因素方差分析，组内两两比较采用*Tukey*检验，*P* < 0.05为差异具有统计学意义。

## 2 结果

### 2.1 3T3-L1前脂肪细胞诱导分化为成熟脂肪细胞

3T3-L1前脂肪细胞呈梭形成纤维细胞形态，胞内无脂滴（图 1A）。诱导分化后，细胞逐渐变圆，胞内开始出现脂滴。随着诱导时间的延长，胞内脂滴逐渐增多，且围绕胞核形成成熟脂肪细胞特征性结构，即“戒环”样结构，表明前脂肪细胞已成功诱导分化为成熟脂肪细胞（图 1B）。油红O染液可将成熟脂肪细胞内的脂滴染成红色（图 1C）。

**1:**
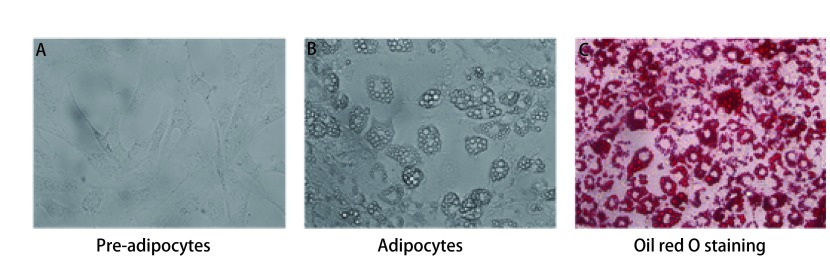
前脂肪细胞的诱导分化及油红O染色。A：3T3-L1前脂肪细胞；B：成熟脂肪细胞；C：成熟脂肪细胞的油红O染色（×400） Differentiation of pre-adipocytes and Oil red-O staining. A: 3T3-L1 pre-adipocytes; B: Mature adipocytes; C: Oil red-O staining of mature adipocytes (×400).

### 2.2 前脂肪细胞与成熟脂肪细胞对A549细胞形态的影响

将肺腺癌A549细胞分别与前脂肪细胞、成熟脂肪细胞共培养2天后，在倒置显微镜下观察细胞形态：空白对照组A549细胞贴壁生长，呈菱形、三角形或鹅卵石样上皮细胞形态（图 2A）。与前脂肪细胞共培养后，A549细胞形态无明显变化（图 2B）。而与成熟脂肪细胞共培养两天后，A549细胞变得更加纤长，呈纺锤样、成纤维样细胞形态（图 2C）。

**2:**
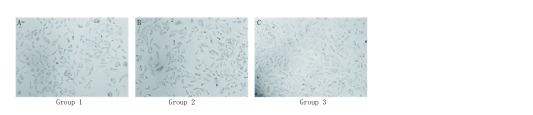
共培养后镜下观察各组A549细胞形态的改变。A: A549细胞单独培养后的细胞形态；B: A549细胞与前脂肪细胞共培养后的细胞形态；C: A549细胞与成熟脂肪细胞共培养后的细胞形态（×100）。 Morphological changes of A549 cells in different groups observed by microscope following co-culture. A: A549 cells morphology after cultivation alone; B: A549 cells morphology after co-cultivation with pre-adipocytes; C: A549 cells morphology after co-cultivation with mature adipocytes (×100).

### 2.3 共培养体系中脂肪细胞可促进A549细胞的增殖

为了评估脂肪细胞在A549细胞生长中的潜在作用，本实验采用了MTT法与平板克隆形成实验检测肺癌细胞增殖率的变化。MTT检测结果显示（图 3A），空白对照组在A549细胞培养24 h、48 h以及72 h后的OD值分别为：（0.309, 8±0.019, 7）、（0.430, 8±0.024, 6）、（0.651, 8±0.031, 9）；前脂肪细胞共培养组分别为：（0.327, 0±0.026, 3）、（0.467, 6±0.027, 8）、（0.606, 0±0.023, 7）；成熟脂肪细胞共培养组分别为：（0.379, 8±0.030, 0）、（0.575, 2±0.039, 2）、（0.855, 6±0.048, 5）。3组A549细胞的生长曲线均呈现出明显的时间依赖性。其中，前脂肪细胞共培养组与空白对照组差异无统计学意义（*P* > 0.05）；而A549细胞与成熟脂肪细胞仅共培养24 h，其增殖速率就明显高于对照组（*P* < 0.01），共培养48 h、72 h后，成熟脂肪细胞的促进作用相较于对照组而言更加明显（*P* < 0.001）。

**3:**
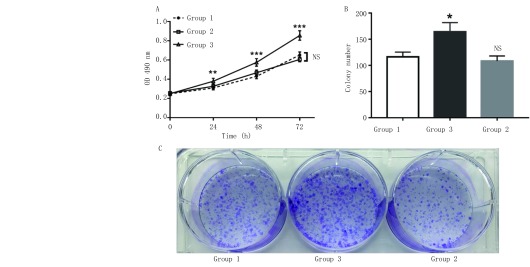
成熟脂肪细胞对A549细胞生长的促进作用。A：MTT法检测结果显示成熟脂肪细胞组中的A549细胞增殖能力明显高于空白对照组与前脂肪细胞组，图表纵坐标表示细胞在波长为490 nm处的吸光度，横坐标表示时间；B-C：平板克隆形成实验：相较于对照组，成熟脂肪细胞可明显促进A549细胞的克隆形成能力，图表纵坐标表示细胞克隆数，横坐标表示相应分组。^*^P < 0.05; ^**^P < 0.01; ^***^P < 0.001 *vs* control。 Mature adipocytespromote the growth of A549 cells. A: MTT assay shows that the proliferation of A549 cells in mature adipocytes group was significantly higher than that in blank control group and pre-adipocytes group, Y-axis represents the absorbance of the cells at wavelength of 490 nm and X-axis represents time period; B-C: Colony formation assay: Compared to the control group, mature adipocytes can significantly promote the cloning ability of A549 cells, Y-axis represents the colony number and X-axis represents thecorresponding groups. ^*^P < 0.05; ^**^P < 0.01; ^***^P < 0.001 *vs* control.

此外，平板克隆形成实验检测共培养两周后各组肺腺癌A549细胞的克隆形成能力，结果显示（图 3B、图 3C），前脂肪细胞共培养组与空白对照组的克隆形成数分别为（115.7±9.2）个、（108.3±10.0）个，此两组差异无统计学意义（*P* > 0.05）；而与成熟脂肪细胞共培养后，A549细胞的克隆形成数为（164.3±17.6）个，明显高于空白对照组（*P* < 0.05）。

### 2.4 共培养体系中脂肪细胞可促进A549细胞的迁移

细胞划痕实验检测A549细胞的横向迁移能力，结果显示（图 4A）：相较于前脂肪细胞共培养组（29.31±5.33）%和空白对照组（26.25±4.12）%，与成熟脂肪细胞共培养24h后的A549细胞的愈合率（47.97±4.26）%明显提高（*P* < 0.05）。Transwell迁移实验检测A549细胞的纵向迁移能力。结果显示（图 4B）：共培养24 h后，空白对照组细胞的穿膜数为（83.6±9.3）个，前脂肪细胞共培养组为（90.2±11.3）个，此两组差异无统计学意义（*P* > 0.05）。而成熟脂肪细胞共培养组的细胞穿膜数为（123.8±14.1）个，明显高于空白对照组与前脂肪细胞组（*P* < 0.01）。统计分析结果显示成熟脂肪细胞在共培养体系中增强了A549细胞的迁移能力。

**4:**
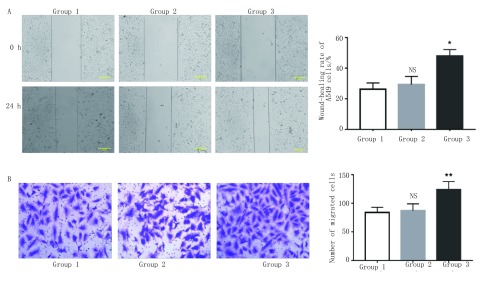
成熟脂肪细胞可促进A549细胞迁移。A：细胞划痕实验（×100）：相较于对照组，成熟脂肪细胞可明显促进A549细胞的横向迁移能力（^*^P < 0.05 *vs* control），图表纵坐标表示细胞划痕愈合率，横坐标表示相应分组；B：Transwell迁移实验（×200）：相较于对照组，成熟脂肪细胞可明显促进A549细胞的纵向迁移能力（^**^P < 0.01 *vs* control），图表纵坐标表示细胞的穿膜数，横坐标表示相应分组。 Mature adipocytes promote the migration of A549 cells. A: Wound-closure assay (×100): Compared to the control group, mature adipocytes can significantly promote the lateral migration of A549 cells (^*^P < 0.05 *vs* control), Y-axis represents the wound-healing rate of cells and X-axis represents the corresponding groups; B: Transwell migration assay (×200): Compared to the control group, mature adipocytes can significantly promote the longitudinal migration of A549 cells (^*^P < 0.01 *vs* control), Y-axis represents the number of migrated cells and X-axis represents thecorresponding groups.

### 2.5 共培养体系中脂肪细胞可促进A549细胞的侵袭

Transwell侵袭实验结果显示（图 5）：共培养24 h后，空白对照组细胞的穿膜数为（76.6±9.2）个，前脂肪细胞共培养组为（69.0±10.7）个，此两组差异无统计学意义（*P* > 0.05）。而成熟脂肪细胞共培养组的细胞穿膜数为（105.2±13.2）个，明显高于空白对照组与前脂肪细胞组（*P* < 0.01）。统计分析结果显示成熟脂肪细胞在共培养体系中增强了A549细胞的侵袭能力。

**5:**
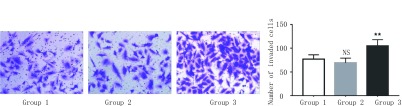
成熟脂肪细胞可促进A549细胞侵袭。Transwell侵袭实验（×200）：相较于对照组，成熟脂肪细胞可明显促进A549细胞的侵袭能力（^**^P < 0.01 *vs* control），图表纵坐标表示细胞的穿膜数，横坐标表示相应分组。 Mature adipocytes promote the invasion of A549 cells. Transwell invasion assay (×200): Compared to the control group, mature adipocytes can significantly promote the invasion of A549 cells (^*^P < 0.01 *vs* control), Y-axis represents the number of invaded cells and X-axis represents thecorresponding groups.

### 2.6 间接共培养下脂肪细胞对肺癌细胞内脂滴含量的影响

在建立共培养模型的基础上培养48 h后，利用油红O染色显示肺癌细胞中脂滴的形态和数量。结果表明：与空白对照组和前脂肪细胞共培养组相比，成熟脂肪细胞共培养组中的A549细胞内聚集了大量脂滴（图 6A）。此外，甘油三酯测定实验的结果与形态学观察结果相一致（图 6B）：与成熟脂肪细胞共培养48 h的A549细胞胞内的甘油三酯含量（0.903±0.095）mmol/g相较于空白对照组（0.501±0.066）mmol/g与前脂肪细胞共培养组（0.476±0.052）mmol/g明显增多（*P* < 0.05）。

**6:**
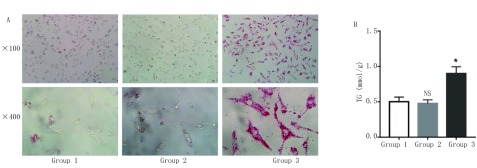
成熟脂肪细胞可促进A549细胞胞内脂质的累积。A：镜下观察共培养后各组A549细胞胞内脂滴的含量；B：成熟脂肪细胞组中的A549细胞的甘油三酯含量明显高于对照组（^**^P < 0.05 *vs* control），图表纵坐标表示细胞内的甘油三酯含量，横坐标表示相应分组。 Mature adipocytes can promote the accumulation of intracellular lipid in A549 cells. A: The lipid level of A549 cells in different groups observed by microscopefollowing co-culture; B: The TG content of A549 cells in mature adipocyte group was significantly higher than that in control group (^**^P < 0.05 *vs* control), Y-axis represents the TG content of cells and X-axis represents thecorresponding groups.

## 3 讨论

肿瘤微环境对肿瘤的发生发展有着十分重要的作用。近年来，已有大量基础性研究证实肿瘤微环境中的脂肪细胞与肿瘤细胞间的相互作用可促进多种恶性肿瘤的演进。在肿瘤细胞的影响下，其附近的脂肪细胞能异常分泌大量脂肪因子（如瘦素、脂联素、内脂素等），生长因子和免疫/炎症/血管生成因子等，这些细胞因子有助于肿瘤细胞的增殖、侵袭、新血管生成、逃避免疫监视和治疗抵抗^[5-9]^。此外，在已被证实为代谢性寄生虫的肿瘤细胞的影响下，脂肪细胞可异常分泌大量代谢底物，如甘油和脂肪酸。这些代谢物可被肿瘤细胞所摄取，用以合成大分子中间产物，并且为肿瘤细胞的增殖、迁移提供所需能量^[14]^。尽管已提出将脂肪细胞与肿瘤联系起来的几种潜在机制，但目前还没有发现关于脂肪细胞及其相关代谢底物在肺癌进展中的作用的确凿证据。

本研究采用Transwell体外共培养模式，探究成熟脂肪细胞对肺腺癌A549细胞生物学行为的影响。通过镜下观察细胞形态，发现与脂肪细胞共培养后的A549细胞由菱形、三角形或鹅卵石样上皮细胞形态向纺锤样、成纤维样细胞形态发展。采用MTT法和平板克隆形成实验检测肺腺癌A549细胞增殖率的变化，结果发现，成熟脂肪细胞能明显促进A549细胞的增殖以及克隆形成能力。通过细胞划痕实验、Transwell迁移与侵袭实验发现，成熟脂肪细胞亦可明显促进A549细胞的迁移与侵袭。而前脂肪细胞对A549细胞的生物学行为无明显影响。前脂肪细胞无论在细胞形态、胞质内容物以及基因表达等方面均与脂肪细胞有着明显的区别，其对乳腺癌的影响目前亦存在争论^[8, 14]^。此外，本研究中使用的3T3-L1前脂肪细胞来源于小鼠胚胎，且考虑到脂肪细胞的功能可因其在体内部位的不同而有一定的差异^[15]^，未来应将人体内的内脏脂肪，皮下脂肪与骨髓脂肪组织应用到此研究中。

肿瘤细胞的糖、脂代谢不同于正常细胞^[16, 17]^。早在20世纪，Warburg等^[18]^发现即使在有氧条件下，肿瘤细胞也以糖酵解途径作为主要代谢方式，称为Warburg效应。此外，Ford等^[19]^研究发现相较于正常细胞，肿瘤细胞的脂肪酸从头合成明显增强。Balaban等研究表明，在乳腺癌MDA-MB-231细胞的影响下，脂肪细胞脂解能力增强，其所大量分泌的甘油与游离脂肪酸被乳腺癌细胞摄取，为乳腺癌细胞提供能量，从而促进乳腺癌的生长与转移^[14]^。本实验中，通过油红O染色以及比色分析实验，我们发现成熟脂肪细胞可促进肺腺癌A549细胞的脂质累积，表明此两组细胞的相互作用所导致的脂质代谢的变化可能与A549细胞生物学行为的改变存在某种联系。在未来的研究中，我们将进一步探索肺癌细胞内所累积的脂质是否源于肿瘤微环境中的脂肪细胞及其对肺癌细胞的能量代谢有何影响。

总之，肿瘤微环境中的脂肪细胞可促进肺腺癌A549细胞的增殖、迁移与侵袭, 且这一促进作用可能与脂质代谢相关。研究肿瘤微环境中的脂肪细胞与肿瘤细胞之间的关系将为更好地明确肿瘤进展的机制提供实验依据，而且有可能成为未来肺癌生物治疗的新靶点。
